# Effects of systemic lidocaine versus magnesium administration on postoperative functional recovery and chronic pain in patients undergoing breast cancer surgery: A prospective, randomized, double-blind, comparative clinical trial

**DOI:** 10.1371/journal.pone.0173026

**Published:** 2017-03-02

**Authors:** Myoung Hwa Kim, Ki Young Lee, Seho Park, Seung Il Kim, Hyung Seok Park, Young Chul Yoo

**Affiliations:** 1 Department of Anesthesiology and Pain Medicine, Yonsei University College of Medicine, 50-1 Yonsei-ro, Seodaemun-gu, Seoul, Republic of Korea; 2 Anesthesia and Pain Research Institute, Yonsei University College of Medicine, 50-1 Yonsei-ro, Seodaemun-gu, Seoul, Republic of Korea; 3 Division of Breast Surgery, Department of Surgery, Yonsei University College of Medicine, 50-1 Yonsei-ro, Seodaemun-gu, Seoul, Republic of Korea; Cliniques Universitaires Saint-Luc, BELGIUM

## Abstract

**Introduction:**

We aimed to compare the effects of intraoperative lidocaine and magnesium on postoperative functional recovery and chronic pain after mastectomy due to breast cancer. Systemic lidocaine and magnesium reduce pain hypersensitivity to surgical stimuli; however, their effects after mastectomy have not been evaluated clearly.

**Methods:**

In this prospective, double-blind, clinical trial, 126 female patients undergoing mastectomy were randomly assigned to lidocaine (L), magnesium (M), and control (C) groups. Lidocaine and magnesium were administered at 2 mg/kg and 20 mg/kg for 15 minutes immediately after induction, followed by infusions of 2 mg/kg/h and 20 mg/kg/h, respectively. The control group received the same volume of saline. Patient characteristics, perioperative parameters, and postoperative recovery profiles, including the Quality of Recovery 40 (QoR-40) survey, pain scales, length of hospital stay, and the short-form McGill pain questionnaire (SF-MPQ) at postoperative 1 month and 3 months were evaluated.

**Results:**

The global QoR-40 scores on postoperative day 1 were significantly higher in group L than in group C (*P =* 0.003). Moreover, in sub-scores of the QoR-40 dimensions, emotional state and pain scores were significantly higher in group L than those in groups M and C (*P* = 0.027 and 0.023, respectively). At postoperative 3 months, SF-MPQ and SF-MPQ-sensitive scores were significantly lower in group L than in group C (*P* = 0.046 and 0.036, respectively).

**Conclusions:**

Intraoperative infusion of lidocaine improved the quality of recovery and attenuated the intensity of chronic pain in patients undergoing breast cancer surgery.

## Introduction

At most, 60% of patients who undergo mastectomy for breast cancer experience chronic pain [1,2], which could deteriorate a patient’s mood, activity, and social function [3,4]. Chronic pain after breast cancer surgery is a significant problem that is expected to become more relevant because the number of patients undergoing breast cancer surgery is increasing owing to the longer survival associated with this surgery.

Many researchers have attempted to improve functional recovery after surgery, as well as acute and chronic pain, with multimodal analgesic methods, including regional and/or systemic analgesia consisting of opioid or other perioperative medications. Several studies have shown that regional analgesia, such as paravertebral block and pectoral nerves block provide better functional recovery or superior pain control after breast cancer surgery [5–7]. However, regional analgesia for breast surgery is not widely used because of its innate risk (e.g., nerve injury or bleeding, especially in patients receiving anticoagulant therapy) and technical challenges. Therefore, easily applicable, safe, and effective alternative analgesic methods are needed.

Recently, perioperative systemic lidocaine and magnesium have been reported to minimize postoperative pain and reduce postoperative morphine consumption [8,9]. However, there has been only one study [10] comparing the effects of systemic lidocaine and magnesium for improving postoperative outcomes, and it focused on relieving acute postoperative pain in patients undergone laparoscopic cholecystectomy.

Therefore, we aimed to investigate the effects of intraoperative systemic lidocaine and magnesium on postoperative functional recovery and chronic pain in patients undergoing mastectomy.

## Methods

This study was a single-center, prospective, double-blind, randomized clinical trial. The protocol was approved by the Institute Research Committee at Severance Hospital, Yonsei University Health System in Seoul, Republic of Korea, 2 July on 2014 (IRB number: 4-2014-0375). This was registered at clinicalTrials.gov (NCT02185859) 7 July on 2014.

### Patients

All adult patients undergoing elective breast cancer surgery at the University Hospital of Yonsei, a tertiary cancer center in Seoul, Republic of Korea, from July 2014 to July 2015 were assessed for eligibility. Written informed consent was obtained from all participants who met the following criteria: an American Society of Anesthesiologists (ASA) physical status of 1-2, aged between 20 and 65 years, scheduled to undergo a mastectomy under general anesthesia before enrollment. Only female patients were enrolled. Patients who had been experiencing pain due to any cause or who were taking analgesics were excluded from this clinical trial. Additionally, patients with a body mass index (BMI) > 30 kg/m^2^, severe heart, kidney, or liver disease, a psychiatric or neurological disorder, contraindications, or allergic responses to lidocaine or magnesium were excluded from participation.

### Interventions

On the morning of the day on which each patient was scheduled for mastectomy, using a random number sequence created by an internet website (*http*:*//www*.*random*.*org*), the patients were randomly allocated to one of three groups in a 1:1:1 ratio: lidocaine group (group L, N = 42), magnesium group (group M, N = 42), or control group (group C, N = 42). The assignments were concealed in a sealed envelope, and randomization was not blocked or stratified. The surgeons, patients, and those assessing outcomes were blinded to the group assignment. A bolus dose of the studied drug was administered for 15 minutes immediately after the subject was brought into the operating room and vital signs were checked from the beginning of anesthesia induction. Subsequently, a maintenance dose of the study drug was continuously administered through the intravenous route, intraoperatively, and was later stopped just before transferring the subject to a recovery room after surgery. Lidocaine (*lidocaine hydrochloride*) and magnesium (*magnesium sulfate*) were administered at 2 mg/kg and 20 mg/kg, respectively, for 15 minutes immediately after induction, followed by infusion at 2 mg/kg/h and 20 mg/kg/h infusion, respectively. Patients in group C were administered and infused with the same volume of saline. The study drugs were prepared by a researcher who was not otherwise involved in the study. Saline was added to the calculated drug doses to achieve a total volume of 50 ml, and the treatments were labeled as “study drug” to ensure double-blinded administration. The concentration of serum magnesium was checked immediately before drug infusion and 1 hour after the infusion was stopped in all study groups. Additionally, we monitored patients closely for any symptoms or signs of possible adverse events associated with lidocaine or magnesium administration, such as electrocardiogram changes during anesthesia, prolonged neuromuscular paralysis, delayed awakening after anesthesia, complaint of a metallic taste, or abrupt change in consciousness, including seizure-like movement postoperatively.

### Clinical manifestations

Upon arrival in the operating room, routine monitoring, including electrocardiography, pulse oximetry, and non-invasive blood pressure, were initiated. Anesthetic depth was monitored using a bispectral index (BIS) monitor (Aspect A-2000^®^, Aspect Medical system Inc., Newton, MA). Patients were administered 0.2 mg glycopyrrolate intravenously, and anesthesia was induced with a bolus administration of 1.5-2 mg/kg of propofol and 1-2 mg/kg of remifentanil; anesthesia was maintained using 4-7% desflurane with an adjuvant infusion of 0.05–0.2 mg kg/min of remifentanil. We adjusted the remifentanil dose to control the blood pressure range to 20% of the baseline blood pressure in all patients. Rocuronium bromide, 0.6 mg/kg, was injected to facilitate tracheal intubation in all patients. Mechanical ventilation was maintained with a tidal volume of 8 ml/kg, and the ventilatory frequency was adjusted to maintain an end-tidal carbon dioxide concentration of 35-40 mmHg with an air/oxygen mixture (fraction of inspired oxygen 0.5). The body temperature was maintained at 36–37°C. In all three groups, the anesthetic depth was titrated to maintain a BIS scores between 40 and 60, and a mean arterial pressure within 20% of the pre-induction values. At approximately 30 min before completion of the operation, 20 mg/kg propacetamol and 0.4 mg/kg nefopam were administered over 10 minutes, and 0.075 mg palonosetron was injected approximately 15 minutes before the end of surgery. Propofol and remifentanil infusions were discontinued upon completion of the surgery, and patients were administered 25 μg/kg neostigmine with 50 μg/kg glycopyrrolate to reverse any residual neuromuscular blockade. When consciousness and spontaneous respiration were adequately restored, the endotracheal tube was removed and the patient recovered for at least 30 minutes in the post-anesthesia recovery unit (PACU). Patients were transferred to the ward and when they met the modified Aldrete scoring system discharge criteria (score ≥ 9 with no score of 1 in any individual category) [11].

### Assessments

#### Quality of recovery 40 survey

A researcher who was unaware of the patients’ group assignments visited each patient to administer the Quality of Recovery 40 surveys (QoR-40) on the day before surgery and on postoperative day (POD) 1, between 6:00 and 8:00 PM. The QoR-40 is used to measure functional recovery and has been validated in patients undergoing general surgical procedures [12]. The global QoR-40 score on POD 1 was the primary endpoint of this investigation. Five general quality-of-recovery dimensions are measured within the QoR-40: physical comfort (12 items), emotional state (9 items), physical independence (5 items), psychological support (7 items), and pain (7 items). Each item is graded on a five-point Likert scale, and the global scores range from 40 (extremely poor quality of recovery) to 200 (excellent quality of recovery). The QoR-40 scoring system was explained in detail to all subjects, completed in the presence of a research assistant, and reviewed to ensure accurate comprehension of all questions.

#### Acute and chronic pain

A 0–10 point numeric rating scale (NRS) was used to measure the degree of pain intensity during each patient’s stay in the PACU and ward. Higher scores indicate a higher degree of pain. Symptoms that the patients complained of besides pain or postoperative nausea and vomiting (PONV), as well as the administered drugs, were investigated, and the length of the stay in the PACU was also recorded. Postoperative opioid consumption (24 hours and 48 hours) was converted to the equivalent dose of intravenous morphine. We assessed the patients’ postoperative chronic pain at 1 month and 3 months after surgery. We checked whether the patients still suffered from the pain, and used the Korean version of short-form McGill pain questionnaire (KSF-MPQ) [13] to measure sensory and affective pain in any patients reporting pain. The KSF-MPQ consists of 17 items, 15 of which are adjectives from the 11 sensory (throbbing, shooting, stabbing, sharp, cramping, gnawing, hot-burning, aching, heavy, tender and splitting) and 4 affective (tiring-exhausting, sickening, fearful and punishing-cruel) categories that are rated on a 4-point intensity scale from 0 (not at all) to 3 (all the time). Sensory categories focus on the nociceptive pain experience, and affective categories focus on the emotional component of nociceptive pain [14]. The other two items assess overall pain intensity: the present pain intensity and a verbal analogue scale score. These two items were excluded in the present study as well as a previous study [13].

### Statistical analyses

Ten-point difference represents a clinically relevant improvement in the quality of recovery based on previously reported values of the mean and range of QoR-40 scores in patients after anesthesia and surgery [15]. With that in mind, the estimated sample size was 37 patients per group with a significance level of 5% (two-tailed), and a power of 90% was achieved when there was a 10-point difference in the QoR-40 on POD 1 among the groups. Therefore, the study sample size was set at 42 patients per group allowing for a dropout rate of up to 10%, resulting in a total of 126 patients. Comparisons were made on an intention-to-treat basis, since it was evident from the results that only the patients who actually received allocated interventions were analyzed.

Continuous variables are expressed as the mean ± standard deviation (SD) (or median [range]), and nominal factors are expressed as n (proportion, %). We performed a one-way analysis of variance (ANOVA) for intergroup comparison of continuous variables. The hypothesis of a normal distribution was confirmed using the Kolmogorov–Smirnov test. Nominal variables, such as the incidence of chronic postoperative surgical pain, are reported as numbers and percentages; these variables were compared among groups by using the Chi-squared test and Fisher’s exact test, as appropriate. Accordingly, the primary outcome (global QoR-40 scores) was assessed using an ANOVA, and the secondary outcome (postoperative pain profiles) was analyzed using ANOVA and chi-square tests. In cases of statistical significance, *post hoc* tests were conducted with Bonferroni adjustment. For statistical analysis, we used SPSS (SPSS INC., Chicago, IL, USA) and considered *P* < 0.05 to be statistically significant.

## Results

**Fig 1:**
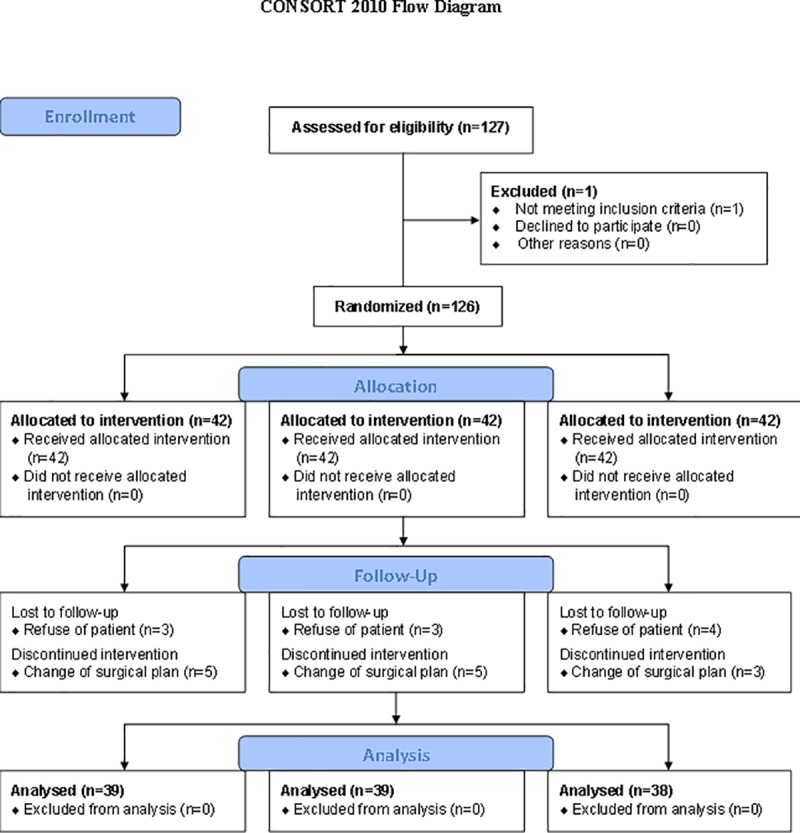
CONSORT 2010 Flow Diagram.

The CONSORT flowchart of present study is shown in Fig 1 Of the 127 patients, who were assessed for eligibility and consented to participate to our study, one patient was excluded because of not meeting inclusion criteria. No one declined to participate to this study. Finally, total 126 patients, who underwent breast cancer surgery (mastectomy) from July 2014 to July 2015, were enrolled in this study. No one declined to this study. Among them, 10 patients (3 patients in group L, 4 patients in group M, and 3 patients in group C) were lost during the follow-up period because of a lack of patient co-operation. Therefore, we collected and analyzed the data from 116 patients (39 patients in group L, 38 patients in group M, and 39 patients in group C) within postoperative 3 months. There were no significant differences among the groups regarding patient characteristics (Table 1). No adverse or unintended effects were observed in the three groups. Table 2 shows the perioperative parameters, where heart rate at extubation was significantly lower in group L than in group C (*P* = 0.003), and the total amount of remifentanil consumption was significantly lower in groups L and M than in group C (*P* < 0.001).

**Table 1:** Patients’ characteristics.

	Group L (n = 39)	Group M (n = 38)	Group C (n = 39)	*P* - value
**Demographic data**				
Age (year)	48.7 (6.4)	48.1 (7.5)	49.0 (6.9)	0.855
Height (cm)	157.6 (3.4)	157.6 (4.1)	157.1 (4.9)	0.840
Weight (kg)	56.4 (3.2)	55.7 (5.2)	55.0 (6.8)	0.530
**ASA physical status**				0.954
1 (number)	33 (84.6%)	31 (81.6%)	32 (82.1%)	
2 (number)	6 (15.4%)	7 (18.4%)	7 (17.9%)	
**Surgical technique**				
Partial mastectomy	26 (66.7%)	22 (57.9%)	27 (69.2%)	
Total mastectomy	13 (33.3%)	16 (42.1%)	12 (30.8%)	
**Lymph node dissection**				0.502
Sentinel	5 (12.8%)	2 (5.3%)	7 (17.9%)	
Axillary	10 (25.6%)	8 (21.1%)	12 (30.8%)	
Sentinel plus Axillary	22 (56.4%)	25 (65.8%)	19 (48.7%)	
**Adjuvant chemotherapy**	15 (38.5%)	14 (35.9%)	15 (39.5%)	0.971
**Adjuvant radiotherapy**	12 (30.8%)	10 (25.6%)	11 (28.9%)	0.903

Values are the mean (SD) or the number of patients (proportion, %), except in the case of age [median (IQR, minimum-maximum)]. SD; standard deviation

C, control; L, lidocaine; M, magnesium; ASA, American Society of Anesthesiologists

**Table 2:** Perioperative parameters.

	Group L (n = 39)	Group M (n = 38)	Group C (n = 39)	*P* - value
**Induction**				
HR (beats/min)	81.8 (12.2)	83.0 (13.9)	82.3 (12.4)	0.934
MAP (mmHg)	90.4 (11.2)	91.1 (12.4)	87.4 (11.5)	0.344
SPO_2_ (%)	98.5 (1.1)	98.4 (1.1)	98.9 (0.7)	0.104
**Extubation**				
HR (beats/min)*	91.3 (10.4)	92.5 (11.6)	98.3 (10.9)	0.013
MAP (mmHg)	94.6 (7.2)	95.7 (12.2)	95.9 (8.7)	0.924
SPO_2_ (%)	100	100	100	1.000
Time (min)	5.8 (0.4)	5.9 (0.3)	6.0 (0.4)	0.208
**Basal Mg (mg/dl)^a^**	0.86 (0.05)	0.87 (0.07)	0.87 (0.07)	0.389
**Postoperative Mg (mg/dl)^a^^†^**	0.86 (0.05)	1.34 (0.05)	0.87 (0.07)	< 0.001
**Remifentanil (μg)^§^**	313.7 (116.4)	374.6 (138.0)	498.4 (150.2)	< 0.001
**Vasopressor (number)**	15 (38.5%)	18 (47.4%)	21 (47.4%)	0.393
**Magnesium (mg)^‖^**	0 (0)	3008.6 (301.3)	0 (0)	< 0.001
**Lidocaine (mg)^¶^**	321.8 (32.0)	0 (0)	0 (0)	< 0.001
**Surgical duration (min)**	102.1 (24.4)	104.5 (34.2)	98.0 (24.2)	0.599
**Anesthetic duration (min)**	127.8 (26.6)	129.5 (35.4)	123.5 (22.5)	0.636
**Administered fluid (ml)**	750.3 (225.5)	792.1 (258.2)	801.4 (204.5)	0.582
**Estimated blood loss (ml)**	29.1 (31.0)	42.5 (68.0)	25.5 (32.7)	0.251

Values are mean (SD) or number of patients (proportion, %).

* Group L *vs*. Group C (*P* < 0.001), Group M *vs*. Group C (*P* = 0.061), Group L *vs*. Group M (*P* = 0.193)

† Group L *vs*. Group C (*P* = 0.967), Group M *vs*. Group C (*P* < 0.001), Group L *vs*. Group M (*P* < 0.001)

§ Group L *vs*. Group C (*P* < 0.001), Group M *vs*. Group C (*P* < 0.001), Group L *vs*. Group M (*P* = 0.154)

‖ Group L *vs*. Group C (*P* = 1.000), Group M *vs*. Group C (*P* < 0.001), Group L *vs*. Group M (*P* < 0.001)

¶ Group L *vs*. Group C (*P* < 0.001), Group M *vs*. Group C (*P* = 1.000), Group L *vs*. Group M (*P* < 0.001)

C, control; L, lidocaine; M, magnesium; HR, heart rate; MAP, mean arterial pressure; SPO_2_, peripheral capillary oxygen saturation

^***a***^ Mg was serum ionized magnesium concentration.

### Primary endpoint

The global QoR-40 scores and the sub-scores of QoR-40 dimensions of the preoperative and POD 1 are presented in Table 3. The preoperative scores of global QoR-40 and sub-scores of QoR-40 dimensions were similar among the three groups. On POD 1, the global QoR-40 score was significantly higher in group L than in group C (*P =* 0.003). In sub-scores of the QoR-40 dimensions, the emotional state and pain scores were significantly higher in group L than in group C (*P* = 0.027 and *P* < 0.001, respectively).

**Table 3:** The global QoR-40 scores and sub-scores of QoR-40 dimensions among the three groups on the preoperative and postoperative day 1.

	Group L (n = 39)	Group M (n = 38)	Group C (n = 39)	*P* - value
**QoR-40 Dimensions**				
**Global QoR-40**				
Preoperative	176.9 (9.4)	175.9 (8.2)	176.4 (10.1)	0.904
Postoperative day 1*	179.3 (6.8)	176.6 (5.6)	173.9 (8.4)	0.005
**Emotional state**				
Preoperative	35.1 (3.5)	34.0 (2.8)	35.0 (4.0)	0.296
Postoperative day 1^†^	38.8 (2.8)	37.1 (1.5)	37.1 (3.4)	0.010
**Physical comfort**				
Preoperative	52.7 (3.6)	52.5 (4.1)	52.8 (4.2)	0.934
Postoperative day 1	55.3 (2.9)	55.4 (2.5)	54.4 (2.9)	0.234
**Psychological support**				
Preoperative	30.8 (1.6)	31.2 (1.8)	30.7 (1.9)	0.347
Postoperative day 1	32.0 (1.5)	32.0 (1.1)	31.7 (1.0)	0.180
**Physical independence**				
Preoperative	24.8 (0.5)	24.7 (0.5)	24.8 (1.3)	0.853
Postoperative day 1	22.3 (0.8)	22.4 (0.9)	22.1 (1.0)	0.452
**Pain**				
Preoperative	33.8 (1.1)	33.7 (1.3)	33.4 (1.1)	0.346
Postoperative day 1^§^	31.3 (1.7)	30.3 (1.6)	29.5 (2.0)	< 0.001

Values are the mean (SD). SD; standard deviation

* Group L *vs*. Group C (*P* = 0.003), Group M *vs*. Group C (*P* = 0.233), Group L *vs*. Group M (*P* = 0.311)

† Group L *vs*. Group C (*P* = 0.027), Group M *vs*. Group C (*P* = 1.000), Group L *vs*. Group M (*P* = 0.023)

§ Group L *vs*. Group C (*P* < 0.001), Group M *vs*. Group C (*P* = 0.101), Group L *vs*. Group M (*P* = 0.050)

C, control; L, lidocaine; M, magnesium; QoR-40, quality of recovery 40

### Secondary endpoint

Outcomes associated with postoperative acute pain are presented in Table 4. At PACU and postoperative 6-24 hours, the pain NRS was significantly lower in groups L and M than in group C (*P* < 0.001 in both groups at PACU, and *P* = 0.001 and 0.046 at postoperative 6-24 hours, respectively). The pain NRS at postoperative 1-6 hours was significantly lower in group L than in group C (*P* = 0.017). However, there were no significant differences in the postoperative intravenous calculated morphine equivalent dose, incidence of PONV, antiemetic consumption at the PACU and during POD 1, and in the length of the hospital stay (Table 4). Table 5 shows postoperative chronic pain. At postoperative 3 months, SF-MPQ and SF-MPQ-sensitive scores were significantly lower in group L than in group C (*P* = 0.046 and 0.036, respectively).

**Table 4:** Postoperative acute pain data.

	Group C (n = 39)	Group L (n = 39)	Group M (n = 38)	*P* - value
**In PACU**				
Pain NRS (0-10)*	2.8 (0.4)	2.2 (0.7)	2.2 (0.5)	< 0.001
Analgesics requirement (number)	11 (28.2%)	10 (25.6%)	11 (28.9%)	0.966
IV ME consumption (mg)	1.7 (3.1)	1.3 (2.2)	1.7 (3.3)	0.758
**Postoperative day 1**				
Pain NRS (0-10)				
Postoperative 6-hour^†^	3.3 (0.9)	2.8 (0.8)	2.9 (0.8)	0.016
Postoperative 24-hour^§^	2.9 (0.7)	2.4 (0.7)	2.5 (0.6)	< 0.001
Analgesics requirement (number)	27 (69.2%)	27 (69.2%)	27 (71.1%)	1.000
IV ME consumption (mg)	5.3 (4.1)	4.6 (4.5)	4.6 (4.2)	0.754

Values are the mean (SD) or number of patients (proportion, %). SD; standard deviation

* Group L *vs*. Group C (*P* < 0.001), Group M *vs*. Group C (*P* < 0.001), Group L *vs*. Group M (*P* = 1.000)

† Group L *vs*. Group C (*P* = 0.017), Group M *vs*. Group C (*P* = 0.298), Group L *vs*. Group M (*P* = 0.758)

§ Group L *vs*. Group C (*P* = 0.001), Group M *vs*. Group C (*P* = 0.046), Group L *vs*. Group M (*P* = 0.601)

C, control; L, lidocaine; M, magnesium; PACU, post-anesthetic care unit; NRS, numeric rating scale; ME, morphine equivalent

**Table 5:** Postoperative chronic pain data.

	Group L (n = 39)	Group M (n = 38)	Group C (n = 39)	*P* - value
**Postoperative 1 month**				
CPSP (number)	6 (15.4%)	7 (18.4%)	10 (25.6%)	0.542
SFMPQ (0-45)	9 (2.1)	9.8 (2.0)	10.6 (2.2)	0.296
SFMPQ-S (0-33)	6.7 (1.5)	7.3 (1.0)	7.8 (1.6)	0.327
SFMPQ-A (0-12)	2.3 (0.7)	2.4 (0.7)	2.7 (0.9)	0.582
**Postoperative 3 months**				
CPSP (number)	7 (17.9%)	8 (21.1%)	14 (35.9%)	0.164
SFMPQ (0-45)*	8.9 (2.3)	10.6 (3.0)	12.7 (2.9)	0.017
SFMPQ-S (0-33)^†^	6.8 (1.7)	7.6 (1.9)	9.8 (2.5)	0.007
SFMPQ-A (0-12)	2.2 (0.7)	3.0 (1.1)	2.9 (0.6)	0.113

Values are the mean (SD) or number of patients (proportion, %). SD; standard deviation

* Group L *vs*. Group C (*P* = 0.046), Group M *vs*. Group C (*P* = 0.110), Group L *vs*. Group M (*P* = 1.000)

† Group L *vs*. Group C (*P* = 0.036), Group M *vs*. Group C (*P* = 0.069), Group L *vs*. Group M (*P* = 1.000)

CPSP, chronic postoperative surgical pain; SFMPQ, short-form *McGill* pain questionnaire; SFMPQ-S, short-form *McGill* pain questionnaire-sensitive; SFMPQ-A, short-form *McGill* pain questionnaire-affective

## Discussion

The present study shows that intraoperative systemic lidocaine improves postoperative quality of recovery, as measured by the QoR-40 survey. Additionally, lidocaine reduced the intensity of chronic pain, and intraoperative opioids requirement in patients undergoing mastectomy for breast cancer. However, intraoperative systemic magnesium was only effective in reducing intraoperative opioid consumption and pain score in the early postoperative period. To our knowledge, the present study is the first to report the effects of systemic lidocaine and magnesium on quality of recovery using the QoR-40 survey and chronic pain with SF-MPQ after breast cancer surgery.

Adequate functional recovery soon after surgery is very important. Traditionally, several parameters including pain, PONV, length of stay in the recovery room, and length of hospital stay are used to estimate postoperative recovery status [16]. Recently, the QoR-40, which was used to measure functional recovery in the immediate postoperative period in this study, has emerged as the only quality of recovery measurement that fulfills the requirements for appropriateness, reliability, validity, responsiveness, precision, interpretability, acceptability, and feasibility [17]. In the present study, when compared to saline infusion, intraoperative systemic lidocaine infusion resulted in significantly higher global QoR-40 scores on POD 1. There was also a significant enhancement in the sub-scores of the emotional state and pain QoR-40 dimensions in patients in the lidocaine group when compared to patients in the control group. Although a previous meta-analysis, which evaluated effects of systemic lidocaine on postoperative recovery after abdominal surgery, concluded that intraoperative lidocaine infusion improves patient rehabilitation and shortens hospital stays [18], there have been few studies conducted on cases of breast cancer surgery. A recent study could not find any favorable effect of intraoperative systemic lidocaine infusion on postoperative recovery, including measures of pain and opioid consumption during the acute postoperative period after mastectomy [19]. However, that study used traditional parameters to measure postoperative quality of recovery rather than the QoR-40. Further, we used 2 mg/kg of lidocaine as a loading dose for 15 minutes immediately after the subject was brought into the operating room, while 1.5 mg/kg of lidocaine was used in the previous study.

Our study showed that both lidocaine and magnesium reduced opioid consumption during surgery and pain intensity during the acute postoperative period. One study, which compared the effects of systemic lidocaine and magnesium on postoperative recovery with traditional parameters for patients undergoing laparoscopic cholecystectomy, reported similar results as ours [10]. However, there has been no study comparing the effects of systemic lidocaine and magnesium in breast cancer surgery. Moreover, a few studies have focused on the effects of systemic lidocaine or magnesium on postoperative pain in breast cancer surgery. For example, a recent meta-analysis, which included only three studies for quantitative analysis, concluded that intraoperative systemic lidocaine infusion had no effect on acute postoperative pain in patients after breast cancer surgery [20]. Although our results showed that intraoperative systemic magnesium had favorable effects on acute postoperative pain control, QoR-40 scores did not improve. *De Oliveira et al*. showed that systemic magnesium enhanced QoR-40 scores at the 24-hour postoperative period in patients undergoing outpatient segmental mastectomy [21]. Considering that the mean postoperative magnesium serum concentration in that study was 1.25 mg/dl and (compared to 1.36 mg/dl in the current study), further studies are needed to investigate the correlation between magnesium serum concentration and its effects on postoperative recovery.

Chronic or persistent postsurgical pain is defined as pain that develops after a surgical procedure that lasts at least 2 months, and where other causes (i.e., malignancy or chronic infection) have been excluded [22]. It has been reported that chronic pain could be associated with postoperative acute pain and the use of analgesics [23], and may be connected with a patient’s emotional aspects [24]. In our results revealed that systemic lidocaine improved not only postoperative acute pain scores, but also emotional state and pain sub-scores of the QoR-40. Moreover, systemic lidocaine also decreased the intensity of chronic pain, which was evaluated using the SF-MPQ at 3 months. The SF-MPQ is the most widely used tool for assessing the quality and intensity of chronic pain [25], and is also a validated model for evaluating chronic pain in Korea [13]. We used all 15 items (11 sensory and 4 affective items) to assess chronic pain in our study. In terms of the effects of lidocaine on chronic pain, our results were similar to those of a past study, which reported the effects of systemic lidocaine on persistent pain after breast surgery [26]. In the present study, although systemic magnesium seemed to decrease the intensity of chronic pain at 3 months compared to saline, there were no significant differences between groups M and C. In a recent retrospective study [27], the perioperative administration of magnesium also did not demonstrate a significant effect on the presence of chronic pain after mastectomy, which is similar to that found in our prospective study.

Inhibition of *N*-methyl-D-aspartate (NMDA) receptors is a common analgesic mechanism of lidocaine [28] and magnesium [29]. However, in our study, systemic liodocaine and magnesium showed different effects on postoperative recovery and chronic pain intensity. Even if we consider that systemic magnesium decreased postoperative acute pain scores and ended to enhance the global QoR-40 on POD 1 and KSF-MPQ at the 3-month postoperative period, only systemic lidocaine had widely favorable effects on early postoperative recovery and chronic pain control. The reason for this discrepancy might be that lidocaine acts via multiple mechanisms for producing analgesia. For example, along with inhibiting NMDA receptors, systemic lidocaine is known to block sodium channels in neurones [30], inhibits G protein-coupled receptors [31], and mitigates neutrophil accumulation and the release of inflammatory mediators [32].

Several limitations of this study should be addressed. First, we did not measure serum lidocaine concentrations. If we had conducted this measurement, the effective dose of lidocaine could have been determined, and the adverse effects of lidocaine could have been monitored more specifically. Nonetheless, the safety of a low-dose lidocaine infusion has been demonstrated in other studies [33,34]. Second, there is a possibility that a larger more bolus dose of magnesium may have more favorable effects, although it remains uncertain whether this would have resulted in a greater magnesium-mediated effect [9]. However, magnesium overdose could be very harmful to patients, and none of the patients who receiving magnesium in the current study experienced adverse effects, such as [35] during magnesium infusion, or any residual neuromuscular paralysis [35]. Third, we excluded the subjects who were suffered from the pain or taking analgesics before surgery. We thought this population would introduce an error in the subject sample in this prospective randomized clinical trial practically. For this reason, we are unable to generalize the effect of lidocaine in all surgical patients having a higher risk of developing chronic postoperative surgical pain, therefore, there is need to investigate all patients including with pain, or those who were taking analgesics before surgery in larger further study. Third, we excluded subjects suffering from pain and/or taking analgesics prior to surgery. We thought this population would introduce an error in the subject sample in this prospective randomized clinical trial, practically. For this reason, we are unable to generalize the effects of lidocaine for all surgical patients including those that have a higher risk of developing chronic postoperative surgical pain; therefore, there is a need to investigate all patients including with those experiencing preoperative pain and those taking analgesics before surgery in a larger further study.

In conclusion, intraoperative systemic lidocaine enhanced postoperative quality of recovery after mastectomy, and improved chronic pain control. While the magnitude of improvement was statistically significant, it is probably less clinically relevant. Therefore, based on this study, we suggest that intraoperative lidocaine administration could be a safe and useful method to manage overall postoperative recovery for patients undergoing mastectomy due to breast cancer.

## Supporting information

S1 File CONSORT 2010 Checklists. (DOC)

S2 File Clinical research protocol (Original language). (DOC)

S3 File Clinical research protocol (English). (DOCX)

S4 File Raw data. (XLSX)
