# Supplementary material for: Effects of systemic lidocaine versus magnesium administration on postoperative functional recovery and chronic pain in patients undergoing breast cancer surgery: A prospective, randomized, double-blind, comparative clinical trial
Source: PLoS One. 2017 Mar 2;12(3):e0173026. doi: 10.1371/journal.pone.0173026 (PMC5333858; doi:10.1371/journal.pone.0173026)
Supplement: S2 File — (DOC) [file pone.0173026.s002.doc]

**Clinical research protocol (Original language)**

**임상연구 계획서**

**1. 연구 제목**

(1) 국문제목: 유방 절제술 환자에서 수술 중 리도카인 정주와 마그네슘 정주가 전신 마취 후 기능적 회복에 미치는 영향

(2) 영문제목: Effect of perioperative intravenous lidocaine infusion and magnesium infusion on the functional recovery after general anesthesia in the patients undergoing breast mastectomy

**2. 연구진 소속, 직위, 성명**

1) 연세 암 병원 마취통증의학과 조교수 유영철

2) 연세 암 병원 마취통증의학과 임상조교수 김명화

3) 연세 암 병원 마취통증의학과 임상조교수 조진선

4) 연세 암 병원 유방암 센터 조교수 박세호

**3. 연구 수행 장소 및 기간**

1) **장소**: 연세대학교 의과대학 연세 암병원

2) **기간**: IRB 승인 일 이후 1 년

**4. 연구 필요성 및 배경**

수술 후 통증은 수술에 따르는 중요한 부작용이고, 이는 각종 합병증의 위험성을 높이며 수술 후의 환자 회복을 지연시킨다.[1] 수술 중에서도 유방 절제술의 경우 술 후 급성 통증뿐 아니라 만성 통증을 환자들이 많이 호소하며, 약 적게는25% 에서 많게는 60% 의 환자에서 수술 후 3개월 후에도 통증이 남아있는 만성 통증 상태 (Chronic Post-Surgical Pain, CPSP)가 된다고 보고되고 있다. 특히 이런 만성 통증 상태인 유방절제술 후 통증 증후군 (Post Mastectomy Pain Syndrome, PMPS) 은 우울증을 야기시키거나 환자의 삶의 질을 떨어뜨리는 중요한 이유가 되지만, 위험 원인 및 발병률이 정확히 알려진 바가 없고 특정 치료도 없는 실정이어서 더욱 환자들이 고통을 받고 있다.[2-5] 그러므로 유방 절제술 환자의 마취에 있어서 수술 후 급성 통증과 만성 통증을 모두 조절할 수 있는 방법이 강구되어야 할 필요가 있다.

많은 수술들에 있어 되도록 적은 용량의 마약성 진통제를 사용하면서 비 마약성 진통제를 추가 하여, 마약성 진통제의 부작용은 줄이면서 수술 후 통증 조절 및 환자의 회복 만족감을 상승시키기 위해 연구 중이다. 이러한 비 마약성 진통제 중에서도 N-methyl-D-aspartate (NMDA) 수용체에 길항제로 작용하는 Lidocaine (리도카인)과 Magnesium (마그네슘) 이 수술적 자극에 대한 통증 과민도를 낮추어 줘서 수술 후 통증 조절에 도움이 된다는 연구 결과가 있어 관심이 되고 있다. [6-8] 수술 중 리도카인의 정주는 수술 후 통증 및 opioid consumption 감소시켜 수술 후 통증조절에 도움을 준다는 보고가 있고, [9] 복강경하 담낭 제거술 환자의 전신마취 이후 수술 후 기능적 회복의 질을 향상시켰다는 보고도 있다. [10] 수술 중 마그네슘 정주의 효과에 대한 연구를 보면 전반적으로 opioid consumption을 감소시키고 수술 후 24 시간 동안의 통증사정도 줄이며, 마그네슘 투여와 관련된 심각한 부작용도 없었기 때문에 마그네슘은 고식적인 opioid-based therapy 에 추가할 만한 진통제로 효과적이라고 하였다. [11] 그러나 수술 중 리도카인과 마그네슘의 정주 중 어떤 약제의 투여가 수술 후 통증 감소에 대한 비교 연구 및 수술 이후 기능적 회복에 도움이 되는지에 대한 연구는 부족하다.

최근 마취의 영역은 마취 이후 회복까지 포함하고 있는데 이러한 회복에 대한 연구의 경향은 어떤 특정 증상의 호전이나 회복 보다는 환자의 전반적인 회복 정도를 측정하여 얼마나 빨리 마취 및 수술 이전의 일상 생활로 복귀할 수 있는가를 측정하는 방향으로 전환되고 있다. 수술 후 회복 (postoperative recovery)은 생리적인 지표 (physiological end-point)나 이상반응 (adverse event)의 발생률 (incidence), 심리적인 상태 (psychological status)의 변화와 같은 다양한 경과와 관련된 복잡한 과정이다.[12] 이를 측정하는 방법으로 가장 널리 사용되고 있는 방법이 Quality of Recovery 40 (QoR-40)이라는 설문 조사이다. 이것은 emotional state(정서 상태), physical comfort(신체적 안정), psychological support(정신적 지지), physical independence(신체적 독립성), pain(통증)의 5가지 세부항목으로 분류 될 수 있는 총 40개의 질문에 대해서 worst 1점, best 5점으로 대답하는 설문으로써, 평균 6.3 분의 시간이 소요되며 test-retest reliability, internal consistency, split-half coefficient가 좋은 설문 방법으로 알려져 있다. [13] 또한 마취 회복과 관련하여 QoR-40은 수술 종류, 마취 방법, 약제의 추가 사용, 성별 등이 마취 및 수술에 미치는 영향을 알아보기 위한 다양한 연구들에서 사용되었고, 이 연구분야에서의 validity도 증명되었다. [14-16]

따라서 본 연구자들은 마취 및 수술 동안의 lidocaine전신적 투여가 QoR40으로 측정한 수술 후의 회복의 질을 향상시킬 수 있을 것이라 가정하였고, 만성 통증인 유방 절제술 후 통증 증후군의 발생율에도 영향을 미칠 것이라 예상하였다.

유방암으로 인해 수술을 받은 환자들의 급성 및 만성 통증에 대해 평가하기 위해 다양한 측정 방법들이 유효성과 신뢰성을 인정받아 사용되고 있지만 그 중에서 short form McGill pain questionnaire (SF-MPQ) 는 classic McGill pain questionnaire 에서 변형된 형태로 sensory, affective와 관련된15 개 질문으로 되어 있고 만성 통증 환자 (암 환자)들에게 더욱 유효성이 인정되는 통증 평가 도구이다.

**5. 위험/이익 분석**

Lidocane의 독성 효과는 혀의 무감각, 심장전도의 이상, 의식 소실이나 경련, 혼수상태 등이 발생할 수 있다고 알려져 있지만 이전의 연구들에서 본 연구와 비슷한 용량으로 lidocaine을 사용하였을 때 특이할 만한 위험증상은 보고된 바 없었다. Magnesium도 근 이완의 효과를 연장시키고 진정 효과도 심화시키며 심장질환이 있는 환자에게 서맥이나 저혈압 등의 악영향을 끼칠 수 있다고는 하지만 역시 이러한 증상들은 독성 효과이며 이전의 연구들에서 본 연구와 비슷한 용량으로 투여하였으나, 역시 관련된 심각한 부작용은 보고된 바 없었다.

오히려 수술 후 통증의 감소에 효과가 있고 마약성 진통제의 양을 줄일 수가 있으므로 마약성 진통제 투여와 관련된 부작용을 감소시켜(호흡억제 및 구역/구토 등) 환자의 만족도를 더 높여줄 것이라 기대하고, 연구 결과 더 효과적인 약제가 있으면 수술 후 진통효과를 위해 적극적으로 사용할 근거를 마련할 수 있다.

**6. 연구 목적**

수술 중 리도카인과 마그네슘의 지속정주가 유방 절제술을 시행 받는 환자에서 전신마취 후 기능적 회복을 도와 궁극적으로 수술 후 회복 개선에 도움을 주는지 알아보고자 한다. 나아가 lidocaine이나 magnesium의 전신 투여와 그에 따른 QoR40의 변화가 유방 절제술 후 만성통증의 발생율에 미치는 영향을 알아보고자 한다.

**7. 목표 대상자의 수 및 산출 근거**

(1) 목표 임상연구 대상자 수: 126명

(2) 산출 근거: QoR-40은 최소 40점에서 최대 200점의 값을 보이며, 대부분의

연구에서 평균 QoR-40의 값의 차이가 10점 이상일 경우 의미 있는 것으로 가정하고 표준편차(SD) 13으로 하고 대상자 수를 산출하였다. alpha 0.05, power 90 %를 바탕으로 위의 가정을 적용하여 산출하면 군당 37명의 환자가 필요하였다. 따라서, 15 %의 case loss를 감안하여 군 당 42명, 총 126명의 환자를 모집하고자 한다. [15]

**Summary Statements**

Group sample sizes of 37 and 37 achieve 90.391% power to reject the null hypothesis of equal means when the populations mean difference is 10.0 with a standard deviation for both groups of 13.0 and with a significance level (alpha) of 0.050 using a two-sided two-sample equal-variance t-test.

**8. 대상자의 선정/제외기준**

(1) 선정 기준: 유방 절제술을 받는 20~65세의 미국마취과의사협회 신체 등급 1 혹은 2에 해당하는 환자

(2) 제외 기준:

① 어떤 원인에서든 기존에 통증이 있는 상태 혹은 진통제를 복용하고 있는 경우

② 임신을 하였거나 임신 가능성이 있는 여자 환자

③ 심각한 심장, 신장 (GFR 이 60 미만), 혹은 간질환이 있는 환자 (ALT와 AST의 합이 100 이상)

④ 정신질환, 신경계질환을 가진 환자

⑤ 리도카인 사용에 금기가 되거나 알러지가 있는 환자

⑥ 마그네슘 사용에 금기가 되거나 알러지가 있는 환자

**9. 대상자 모집 방법 및 동의 절차**

마취 전 평가실 (외래)에서 또는 수술 하루 전에 시행하는 술 전 방문 시에 대상자에게 연구의 목적과 방법에 대해 설명하고, 연구 참여에 대해 동의서를 받는다. 설명 및 동의서 취득은 독립된 공간에서 시행하며 설명으로부터 동의를 결정하기까지의 시간은 충분히 주도록 한다. 연구참여 동의의 지속 여부를 연구 기간 중에 시행되는 여섯 차례의 방문 때 마다 (수술 전날, 수술실, 회복실, 수술 1 일 뒤, 수술 1 개월 뒤, 수술 3 개월 뒤) 구두로 확인한다.

취약한 대상자 모집 시, 보호 방안: 강제력이나 부당한 영향에 취약하기 쉬운 연구 참여자 또는 의사결정능력이 결여된 취약한 대상자들은(임부, 소아나 미성년자, 의사결정능력이 결여된 자, 자발적 참여 결정에 영향을 받기 쉬운 자 등) 임상연구 대상에 포함되지 않거나 선정에서 제외된다.

**10. 스크리닝 검사 항목 및 방법**

① 환자의 과거력과 내, 외과적 기왕력

② 수술 전 흉부 방사선 검사, 말초혈액검사, 전해질검사, 혈당검사, 소변검사

- 스크리닝 검사항목은 수술 전 일반적으로 시행하는 것으로, 이 연구를 위해 추가로 시행하는 검사는 없다.

**11. 연구 설계 및 방법**

(1) 본 연구는 전향적 무작위 이중 맹검 조절연구 (Prospective Randomized Double-Blind Controlled Study)이다.

(2) 유방 절제술을 받을 예정인 환자들을 수술 전날에 병동 상담실 혹은 외래 수술실 내 상담실에서 연구의 목적과 방법에 대해 설명하고 수술 당일 연구에 대해 이해하고 동의하는 환자들에 한해 연구 참여에 대한 동의서를 받는다. 동의서 작성 후 모든 대상자자들은 QoR-40 설문지를 작성한다. 연구 참여 동의 획득 및 술 전후 평가는 한 명의 연구자가 시행하고, 이 연구자는 수술 중 마취 및 연구약제 투여에 관여하지 않으며, 대상자 개개인의 배정 군을 알지 못한다.

(3) 연구 책임자는 모든 대상자들을 www.randomizer.org/form.htm에서 제공된 무작위 배정표에 따라 대조군 (group C) 혹은 Lidocaine군 (group L), Magnesium 군 (group M)으로 배정한다. 배정된 군에 따른 투여 약제들은 연구 책임자가 준비한다. (Group C, 50 cc syringe에 0. 9 % normal saline 40 mL 준비; Group L, 50 mL syringe에 1 % lidocaine 40 mL (400mg) 준비; group M, 50 mL syringe에 MgSO4 4g 40ml 준비)

(4) 모든 연구 대상자에 대해 마취 및 회복은 배정 군을 알지 못하고 수술 전후 평가에 참여하지 않는 또 다른 연구자가 담당하게 된다. 연구 약제의 투여를 제외하고는 통상적으로 연세 암병원에서 유방 절제술을 받는 환자에서의 마취와 동일하게 관리한다.

대상환자가 수술실에 도착하면 심전도와 맥박산소계측기를 부착하고 혈압을 측정한다. 마취 유도를 위해 propofol 1.5 ~ 2.0 mg/kg 투여 및 remifentanil 0.2mcg/kg/min 을 지속정주하고, rocuronium 0.6 mg/kg으로 근 이완 후 기관 내 삽관을 시행한다. 마취 유지는 50% 산소, desflurane, remifentanil로 한다. 적절한 마취 깊이를 유지하기 위해 bispectral index를 감시하며 bispectral index score를 40~60 정도로 유지할 수 있게 desflurane의 투여 농도를 조절한다. 또한 피험자의 기저 혈압 및 맥박수의 20% 범위에서 수술 중 혈압 및 맥박수가 유지될 수 있도록 remifentanil infusion의 속도는 0.05~0.5mcg/kg/min 범위에서 조절한다. 수술 예상 종료 시점 30 분 전에 수술 후 통증 조절을 위해 propacetamol 2g을, 구역 구토 예방을 위해 palonosetron 0.075mg을 정주한다. 수술이 끝나면 desflurane과 remifentanil의 투여를 종료하고, 신경 자극기를 사용하여 사연속 자극 (train of four, TOF)을 시행하여 근 수축을 평가한 뒤, glycopyrrolate 0.2 mg과 neostigmine 1 mg으로 근이완을 역전 시킨다. 대상환자의 의식이 돌아오고 호흡이 회복되면 발관하고 회복실로 옮긴다.

(5) 연구약제들은 대상자가 수술에 입실하고 마취 유도 후부터 bolus 용량을 15 분간 투여하고 이어서 maintenance 용량을 지속 정주하다가 대상환자를 extubation (발관) 직후 회복실로 옮기기 직전 투여를 중단한다. (Group L의 대상자의 경우 마취 유도 직후 lidocaine 2 mg/kg를 15분간 투여 받고, 이어서 2 mg/kg/h로 지속 투여 받는 것이고, group M 의 대상자의 경우 20 mg/kg를 15분간 투여 받고, 20mg/kg/h로 지속 투여 받는 것이며, group C 대상자의 경우 같은 용적의 생리식염수를 투여 받는 것이다. 본 연구의 lidocaine 및 magnesium의 투여 용량은 이전의 연구에서 안전성과 진통효과가 입증된 바 있다[17]). 그러나 총 마취 시간이 4시간이 넘어가게 되면 연구약제의 투여를 중단하여 과도한 투여를 예방한다.

(6) 환자의 회복실 및 수술 이후의 관리로 적어도 리도카인이나 마그네슘의 작용시간이 끝나는 시점까지 이상반응에 대한 모니터링이 필요하다. 따라서 리도카인과 관련된 이상반응 및 독성효과에 대한 감시로 마취 종료 후 리도카인 투여를 중단하고 나서부터 회복실에 있는 동안에 환자의 심전도, 호흡수, 산소포화도 등의 활력징후를 모니터링 하면서 발음이 어눌해지거나 의식의 변화가 없는지 확인하고 30 분 이상 관찰한 뒤 이상이 없으면 퇴실하게 된다. 마그네슘의 이상반응 및 독성효과에 대한 감시 계획 또한 마그네슘 투여를 중단하고 나서 회복실에 있는 동안에 기본적인 활력징후를 관찰함은 물론 슬개건 반사를 확인하여 근육 수축에 문제가 없는지도 확인하며 30 분 이상 관찰한 뒤 이상이 없으면 퇴실하게 된다.

그리고 수술1 일 뒤에 QoR 40 설문을 위해 환자를 방문할 때 환자에게 지연되어 나타날 수 있는 리도카인이나 마그네슘 이상 반응 및 독성 반응도 같이 확인한다.

(7) 마그네슘 군에서 안전한 범위 내에 마그네슘이 혈중에 분포하고 있는지, 그리고 마그네슘 군에서 효과가 있다는 결과가 나온다면 마그네슘의 혈중 농도가 어느 정도일 때 인지를 알아보기 위해서 마취 유도 시 연구 약제 투여 직전에 배정된 군과 상관없이 모든 환자들의 기저 마그네슘 혈중 농도를 측정하고 연구 약제 투여 중단 후 2 시간 뒤에도 측정한다.

(8) 연구 약제의 관리: 연구 약제의 보관 및 관리는 연구책임자 (유영철) 혹은 연구책임자가 지정한 연구담당자 (김명화)가 할 계획임.

**11. 대상질환의 표준치료 방법**

일반적으로 유방 절제술 이후 통증 조절은 수술 직후에는 fentanyl이나 propacetamol 을 투여하고, 수술 후 병실에서는 tramadol 을 필요 시 정주 방법 (PRN) 으로 주로 투여하고 있다. 그런데 Fentanyl 과 같은 마약성 진통제는 용량이나 환자 상태에 따라 구역/구토, 호흡수 저하 등의 부작용이 일어날 수 있다.

**12. 유효성 평가항목 및 방법**

(1) 환자의 배정 군을 모르는 다른 연구자가 수술 전, 수술 후 1일 째 에 환자를 방문하여 QoR-40 질의를 한다. 정서상태(Emotional status), 신체적 안정(Physical comfort), 정신적 지지(Physiological support), 신체적 독립성(Physical independence), 통증(Pain)의 다섯 가지 항목에 대한 Subtotal score와 합산된 Global score (Total 200점)로 환자의 전신마취 후 회복의 질을 평가한다. 수술 24시간 후의 QoR 40이 이 연구의 primary end point이며, 두 군 간에 평균 10 점 이상의 차이가 있을 때 군 간 유의한 차이가 있는 것으로 한다.

(2) 마취 유도 전 처음 측정된 활력징후를 기록한다.

(3) 수술 중 BIS를 40-60으로 유지하는데 필요했던 과 혈압과 맥박을 baseline의 20% 범위 안으로 유지하는데 필요했던 desflurane 의 평균 농도 및 remifentanil의 총 사용량을 기록한다.

(4) 수술 종료 후,

① 수술 시작부터 종료까지 총 수술시간, 마취 유도부터 투여 중단한 시점까지를 총 마취 시간으로 한다.

② 회복의 속도를 측정하기 위해 주 마취제의 투여를 중단한 시점으로부터 구두 명령에 반응하기까지 걸린 시간 및 BIS 점수, 기관내관을 발관하기까지 걸린 시간 및 BIS 점수를 기록한다. 각 시간에 활력징후도 평가한다.

③ 마취 유지 중 50 회/분 미만의 서맥이나, MAP < 60 mmHg 미만의 저혈압을 보인 시간과 사용된 약제를 기록한다. 그 외 발생한 문제들에 대해서도 평가하고 사용한 약제를 기록한다.

(6) 회복실 입실 후,

① 환자의 통증 정도를 측정하기 위해 입실 시와 퇴실 시에 각각 0-10 점으로 나타내는 Numeric Pain Intensity Scale (NPIS)를 사용하여 평가하며, 아플수록 점수가 높다.

② 회복실에서 사용한 진통제 및 항 구토제를 기록하고 회복실 입실부터 퇴실까지의 체류시간을 기록한다.

③ 진통이나 구역/구토 외에 환자가 호소한 다른 증상들 및 투여한 약제들도 기록한다.

(7) Sedation score(진정 정도)는 회복실 입실 및 퇴실 시에 시행하며 5 point scale을 이용한다. [18]. 1-5 점의 점수로 환자의 진정 상태를 평가하며 1점은 완전히 깨어있을 때, 2 점은 약간 졸리운 상태, 3 점은 자는 듯하지만 목소리에 반응하는 정도, 4점은 자는 양상 보이며 목소리에 속도도 느리게 반응할 때, 5 점은 통증 같은 자극에는 반응하지만 목소리에는 반응하지 않고 자는 모습을 보일 때를 말한다.

(8) 수술 후 24 시간 뒤 각각 진행되는QoR 40 측정 시점에 그때까지 투여된 진통제의 종류 및 총 사용량을 기록한다. 또한 수술 후 QoR 40 측정 시점에 그 때까지 발생한 구역 구토의 횟수, 정도와 항 구토제의 사용량을 기록하며, 피험자의 수술 후 퇴원까지의 재원기간도 확인하여 기록한다.

(9) 수술 종료 24시간의 급성 통증 정도 및 양상의 평가 – Numeric Pain Intensity scale (NPI scale), short form McGill pain questionnaire (SF-MPQ)

(10) 수술 1개월 후 술 후 통증의 유무, 통증이 있다면 통증의 위치 및 양상에 대한 평가

: SF - MPQ

(11) 수술 3개월 후 술 후 만성 통증의 유무, 통증 있다면 통증 위치 및 양상의 평가

: SF - MPQ

(12) 결측치의 해결 방법: 이 연구는 환자에게 설문을 하는 등의 환자 순응도와 관련된 부분이 크므로 결측치 발생 시 대상 환자의 자료는 분석에서 제외하도록 한다.

**13. 통계분석 방법**

① 유의수준 5% 에서 모든 가설에 대한 검정을 시행하고 원칙적으로 양측 검정을 한다.

② 모든 연속형 변수는Mean ± SD (혹은 Median[IQR] 로 표현하며 모든 명목 변수는 n(%)로 표현한다.

③ QoR 40의 군 간 비교 (QoR40 및 SF-MPQ)는 평균값에 대해 One-way ANOVA를 시행한다. 그 이외의 관찰 변수인 수술 중 desflurane 농도, remifentanil의 사용량, 회복시기에 측정한 각종 시간 지표, 수술 후 병원 재원 기간, 수술 후 진통제 사용량, 구역/구토의 횟수, 항 구토제 사용량 등의 변수도 모두 연속변수이므로 One-way ANOVA를 시행한다.

④ 환자의 과거력, ASA score 등과 같은 순위형 및 명목형 변수의 경우 Chi-square와 Fisher’s exact test를 통해 비교하고, 시간에 따른 통증 및 진정 정도, 회복 정도를 나타내는 수치의 의미 있는 변화를 알아보기 위해 반복 측정된 연속 변수이므로 *post hoc* 분석을 통해Tukey’s test와 Bonferroni’s correction을 시행한다.

- 통계분석에는 SPSS (SPSS Inc., Chicago, IL, USA)를 사용하며, P < 0.05 일 때 통계적으로 의미 있는 것으로 간주한다.

**14. 임상연구 중지·탈락 기준**

(1) 대상환자가 연구 참여를 거부한 경우: 동의서를 받은 이후라도 환자의 마취 전 및 마취 후 방문 시에 연구에의 참여 의사를 다시 확인하여, 환자가 언제든 참여 철회 의사를 연구진에게 밝힐 수 있도록 한다.

(2) 수술 후, 수술 관련 합병증 또는 전이 등의 이유로 입원 기간 중 재수술을 시행한 환자

**15. 예측 부작용 및 사용상의 주의사항**

**Lidocaine**은 드물게 가려움이나 피부 발진 등의 알러지 반응이 나타날 수 있고, 오심이나 구토 등의 증상도 나타날 수 있다. 또한 과량 투여 시 독성 효과 (toxic effect)가 나타날 수도 있는데, [19] Lidocane의 독성 효과는 혈중농도 5-10 µg/mL에서 어지러움이나 혀의 무감각이, 10-15 µg/mL 에서 의식 소실이나 경련, 15 µg/mL 이상에서 혼수상태가 발생할 수 있다고 알려져 있다. 하지만 이런 독성 효과는 혈중 농도가 5 µg/mL 이상일 때 나타나며 lidocane 400 mg을 일시에 투여해야 하는 것으로 알려져 있다. 그러나 이전의 연구들에서 본 연구와 비슷한 용량으로 lidocaine을 투여하면서 다양한 시점에서 혈중 lidocaine 농도를 측정한 결과에서도 5 µg/mL를 넘은 것은 없었다. [20]

리도카인과 관련된 이상반응 및 독성효과에 대한 감시계획은 마취 종료 후 리도카인 투여를 중단하고 나서부터 회복실에 있는 동안에 환자의 심전도, 호흡수, 산소포화도 등의 활력징후를 모니터링 하면서 발음이 어눌해지거나 의식의 변화가 없는지 확인하고, 수술 1 일, 2 일 뒤에도 환자를 방문하여 이상 증상이 없는지 확인하는 것이다.

리도카인 이상 반응에 대한 치료 계획은 closed monitoring 이 원칙이며, 심한 독성 반응으로 경련이 나타나면 항경련제를 투여하고, 의식소실이나 혼수 등의 심한 의식변화에 의한 호흡 부전에 대해서는 빠른 기도 확보 및 호흡 보조를 해 주는 것이다.

**Magnesium**은 오심이나 구토, 설사 등의 위장관 관련 증상이 나타날 수 있고, 혈청 마그네슘 농도가 4 mEq/L 이상일 때는 독성 효과가 나타나기도 한다. 대표적인 독성효과로 흔히 근이완의 효과가 연장되고 진정 효과를 심화시키게 되며, 심장질환이 있는 환자에게 심한 서맥이나 저혈압 등의 악영향을 끼칠 수 있다. [21-23] 그러나 이전의 연구들에서 본 연구와 비슷한 용량으로 투여하였을 때 혈청 농도는 4 mEq/L 를 넘지 않았고, 심각한 부작용의 보고는 없었다.

마그네슘의 이상반응 및 독성효과에 대한 감시 계획은 마그네슘 투여를 중단하고 나서 회복실에 있는 동안에 기본적인 활력징후를 관찰하는 것은 물론 슬개건 반사를 확인하여 근육 수축에 문제가 없는지 확인하고, 수술 1 일과 2 일 뒤에도 환자를 방문하여 이상 증상의 유무를 확인하는 것이다. 그리고 연구 약제 투여의 중단 후 2 시간 뒤에 마그네슘 혈 중 농도를 측정한다.

고마그네슘혈증 및 독성효과에 대한 치료계획은 신경근 기능과 심혈관 효과를 길항하기 위해 칼슘글루콘산염을 정주하거나, 수액 투여 및 furosemide 이뇨제를 투여하여 마그네슘 수치를 감소시키는 방법이 있다. 만일, 심한 독성반응으로 인해 호흡근의 약화로 호흡부전이 발생하게 되면 기도 확보 및 호흡보조가 필요할 수도 있다.

**16. 부작용을 포함한 안전성의 평가기준, 평가방법 및 보고방법**

대상환자 모두에 대하여 마취 유도 전, 마취 유지 중, 기관 발관 후 회복실 까지 숙련된 마취통증의학과 전문의의 감시 하에 혈역학적 안정성과 적절한 환기여부를 평가하고 전문의의 결정에 따라 심혈관계 보조 약물 투여, 호흡 보조, 흡입 산소 분율 증가 등의 처치를 한다.

알레르기 반응을 포함하여 예상하지 못한 약물의 부작용이 발견될 경우 즉시 적절한 처치를 하고 1주일 이내에 IRB에 보고 한다.

또한, 임상시험기간 중 다음의 하나 또는 그 이상에 해당하는 뜻밖의 의학적 발생 (사망한 경우, 생명을 위협하는 경우, 입원 또는 입원기간의 연장이 필요한 경우, 지속적 또는 의미 있는 불구나 기능 저하를 초래하는 경우, 의학적으로 중요한 사건) 시에는 이를 인지한지 24시간 이내에 시험자와 연구자는 반드시 IRB에 보고하도록 하며, SUSAR 발생 시 식약청에 보고하도록 한다. 모든 중대한 이상 반응에 대한 정보는 이상반응 양식에 이상반응에 대한 정보와 추가적인 추적 정보를 기재할 것이며, 보고의 사본은 피험자의 CRF와 함께 반드시 보관되도록 한다.

**17. 개인정보 보호 및 연구자료의 기밀 유지를 위한 방안**

연구 자료는 본 연구를 위한 목적으로만 사용되며 연구책임자, 연구담당자 외에 자료의 열람과 분석을 제한한다. 임상연구 대상자의 개인식별 정보는 식별정보는 코드화하여 screening sheet와 분리하여 기록한다. 자료는 잠금 장치가 있는 장과 접근이 제한된 컴퓨터에 보관하도록 한다.

**18. 중간분석에 대한 계획 및 필요 시 연구의 조기 종료하는 범위를 포함하여 연구의 통계분석에**

**대한 계획 기술**

- 중간 분석에 대한 계획 및 조기 종료에 대한 계획은 없다.

**19. 지속적인 안전성 모니터링 계획 및 자료안전모니터링계획**

연구 책임자(연구 책임자 부재 시 위임 받은 연구 담당자)를 자료안전담당자로 하여 월 1 회 연구

자료를 모아 검토하고 관리하여 지속적인 안전성에 대한 모니터링을 시행하여 자료의 완전성을

보증하고 연구 대상자의 안정성을 확보한다.

**20. 연구실행 계획표(일정표)**

(1) 연구 계획 수립 및 IRB 승인: 2014년 5월 1일 ~ 2014년 6월 30일 (예정)

(2) 연구 대상자 모집 및 연구 진행: IRB 승인 후 1년

(3) 연구 자료 통계 처리 및 결과 분석: 대상자 모집 완료 후 2개월

(4) 논문 작성: 결과 분석 후 3개월

**21. 참고문헌**

1. Wu CL, Raja SN: **Treatment of acute postoperative pain**. *Lancet* 2011, **377**(9784):2215-2225.

2. Alves Nogueira Fabro E, Bergmann A, do Amaral ESB, Padula Ribeiro AC, de Souza Abrahao K, da Costa Leite Ferreira MG, de Almeida Dias R, Santos Thuler LC: **Post-mastectomy pain syndrome: incidence and risks**. *Breast* 2012, **21**(3):321-325.

3. Meijuan Y, Zhiyou P, Yuwen T, Ying F, Xinzhong C: **A retrospective study of postmastectomy pain syndrome: incidence, characteristics, risk factors, and influence on quality of life**. *ScientificWorldJournal* 2013, **2013**(1537-744X (Electronic)):159732.

4. Fecho K, Miller NR, Merritt SA, Klauber-Demore N, Hultman CS, Blau WS: **Acute and persistent postoperative pain after breast surgery**. *Pain Med* 2009, **10**(4):708-715.

5. Jain G, Bansal P, Ahmad B, Singh DK, Yadav G: **Effect of the perioperative infusion of dexmedetomidine on chronic pain after breast surgery**. *Indian J Palliat Care* 2012, **18**(1):45-51.

6. Fawcett WJ, Haxby EJ, Male DA: **Magnesium: physiology and pharmacology**. *Br J Anaesth* 1999, **83**(2):302-320.

7. Kehlet H, Wilmore DW: **Multimodal strategies to improve surgical outcome**. *Am J Surg* 2002, **183**(6):630-641.

8. Koppert W, Weigand M, Neumann F, Sittl R, Schuettler J, Schmelz M, Hering W: **Perioperative intravenous lidocaine has preventive effects on postoperative pain and morphine consumption after major abdominal surgery**. *Anesth Analg* 2004, **98**(4):1050-1055, table of contents.

9. Vigneault L, Turgeon AF, Cote D, Lauzier F, Zarychanski R, Moore L, McIntyre LA, Nicole PC, Fergusson DA: **Perioperative intravenous lidocaine infusion for postoperative pain control: a meta-analysis of randomized controlled trials**. *Can J Anaesth* 2011, **58**(1):22-37.

10. De Oliveira GS, Jr., Fitzgerald P, Streicher LF, Marcus RJ, McCarthy RJ: **Systemic lidocaine to improve postoperative quality of recovery after ambulatory laparoscopic surgery**. *Anesth Analg* 2012, **115**(2):262-267.

11. Albrecht E, Kirkham KR, Liu SS, Brull R: **Peri-operative intravenous administration of magnesium sulphate and postoperative pain: a meta-analysis**. *Anaesthesia* 2013, **68**(1):79-90.

12. Myles PS, Hunt JO, Nightingale CE, Fletcher H, Beh T, Tanil D, Nagy A, Rubinstein A, Ponsford JL: **Development and psychometric testing of a quality of recovery score after general anesthesia and surgery in adults**. *Anesthesia and analgesia* 1999, **88**(1):83-90.

13. Myles PS, Weitkamp B, Jones K, Melick J, Hensen S: **Validity and reliability of a postoperative quality of recovery score: the QoR-40**. *Br J Anaesth* 2000, **84**(1):11-15.

14. Murphy GS, Szokol JW, Greenberg SB, Avram MJ, Vender JS, Nisman M, Vaughn J: **Preoperative dexamethasone enhances quality of recovery after laparoscopic cholecystectomy: effect on in-hospital and postdischarge recovery outcomes**. *Anesthesiology* 2011, **114**(4):882-890.

15. De Oliveira GS, Jr., Ahmad S, Fitzgerald PC, Marcus RJ, Altman CS, Panjwani AS, McCarthy RJ: **Dose ranging study on the effect of preoperative dexamethasone on postoperative quality of recovery and opioid consumption after ambulatory gynaecological surgery**. *Br J Anaesth* 2011, **107**(3):362-371.

16. Buchanan FF, Myles PS, Cicuttini F: **Effect of patient sex on general anaesthesia and recovery**. *Br J Anaesth* 2011, **106**(6):832-839.

17. Kuo CP, Jao SW, Chen KM, Wong CS, Yeh CC, Sheen MJ, Wu CT: **Comparison of the effects of thoracic epidural analgesia and i.v. infusion with lidocaine on cytokine response, postoperative pain and bowel function in patients undergoing colonic surgery**. *British Journal of Anaesthesia* 2006, **97**(5):640-646.

18. Paris A, Kaufmann M, Tonner PH, Renz P, Lemke T, Ledowski T, Scholz J, Bein B: **Effects of clonidine and midazolam premedication on bispectral index and recovery after elective surgery**. *Eur J Anaesthesiol* 2009, **26**(7):603-610.

19. Liu S: **Local Anesthetics: Clinical Aspects**. In: *Essentilas of Pain Medicine and Regional Anesthesia.* Edited by HT B, SN R, RE M, SS L, SM F. Philadelphia, PA, USA: Elsevier, Churchill Livingstone; 2005: 558-565.

20. McCarthy GC, Megalla SA, Habib AS: **Impact of intravenous lidocaine infusion on postoperative analgesia and recovery from surgery: a systematic review of randomized controlled trials**. *Drugs* 2010, **70**(9):1149-1163.

21. Fuchs-Buder T, Wilder-Smith OH, Borgeat A, Tassonyi E: **Interaction of magnesium sulphate with vecuronium-induced neuromuscular block**. *Br J Anaesth* 1995, **74**(4):405-409.

22. Vissers RJ, Purssell R: **Iatrogenic magnesium overdose: two case reports**. *J Emerg Med* 1996, **14**(2):187-191.

23. Kiran S, Gupta R, Verma D: **Evaluation of a single-dose of intravenous magnesium sulphate for prevention of postoperative pain after inguinal surgery**. *Indian J Anaesth* 2011, **55**(1):31-35.
