# Supplementary material for: Effects of systemic lidocaine versus magnesium administration on postoperative functional recovery and chronic pain in patients undergoing breast cancer surgery: A prospective, randomized, double-blind, comparative clinical trial
Source: PLoS One. 2017 Mar 2;12(3):e0173026. doi: 10.1371/journal.pone.0173026 (PMC5333858; doi:10.1371/journal.pone.0173026)
Supplement: S3 File — (DOCX) [file pone.0173026.s003.docx]

**Clinical research protocol (English)**

**Clinical research protocol**

**1. Title**

Effect of perioperative intravenous lidocaine infusion and magnesium infusion on the functional recovery after general anesthesia in the patients undergoing breast mastectomy

**2. Institution and study period**

(1) Institution: Yonsei University College of Medicine, Severance Hospital

(2) The period: 1 year within IRB certification

**3. Backgrounds**

Postsurgical pain is a relevant side effect following surgery which increases the risk of various complications and delays postoperative patient recovery. [1] Among the many types of surgeries, mastectomy causes not only acute but also chronic pain in many patients. It was reported that 25 to 60% of mastectomy patients experienced chronic postsurgical pain (CPSP), which is defined as pain remaining three months after surgery. In particular, postmastectomy pain syndrome (PMPS), a CPSP state, causes depression and serves as a critical factor in decreasing a patient’s quality of life. However, because the risk factors and the incidence rate are not accurately known and there is not a specific treatment available, many patients are suffering from PMPS. [2-5] Therefore, it is necessary to find a treatment method that controls both postsurgical acute pain and postsurgical chronic pain during anesthesia of a mastectomy patient.

Lidocaine is a commonly used amide type local anesthetic, but it is also used for general administration as an adjuvant therapy to decrease postsurgical pain due to its analgesic, anti-hyperalgesic, and anti-inflammatory properties. Systemic administration of lidocaine has been reported to reduce postsurgical pain and the analgesic dose mainly in abdominal and thoracic surgeries. [6-8] In particular, reduced pain as well as a decreased length of stay from a decreased ileus period has been reported in abdominal surgeries, [9] showing the effect of lidocaine in facilitating postsurgical recovery. [10] However, there are no sufficient studies on the effect of systemic lidocaine administration to enhance recovery in other types of surgery performed on other body parts. In addition, a review article on the effect of intraoperative intravenous injection of magnesium found it to be an effective analgesic that may be added to conventional opioid-based therapy because it generally reduces opioid consumption, decreases pain assessment for 24 hours after surgery, and lacks severe side effects in relation to magnesium administration. [11] However, there has been insufficient research comparing the intraoperative intravenous injection of lidocaine or magnesium in terms of which is more helpful for general functional recovery and decreased postoperative pain.

Recently, the scope of research on anesthesia has come to embrace postanesthetic recovery; to help patients return to daily life, the research trend is now shifting from the improvement or resolution of a specific symptom to the measurement of general recovery. [12] A widely used method to measure postoperative recovery is the Quality of Recovery 40 (QoR-40) survey. Five general quality-of-recovery dimensions are measured within the QoR-40: physical comfort (12 items), emotional state (9 items), physical independence (5 items), psychological support (7 items), and pain (7 items). Each item is graded on a five-point Likert scale, and the global scores range from 40 (extremely poor quality of recovery) to 200 (excellent quality of recovery). The QoR-40 scoring system was explained in detail to all subjects, completed in the presence of a research assistant, and reviewed to ensure accurate comprehension of all questions. [13] QoR-40 now remains the only quality of recovery measurement that fulfills the requirements for appropriateness, reliability, validity, responsiveness, precision, interpretability, acceptability, and feasibility. [14-16]

On the other hand, CPSP is a pain syndrome that may present following surgery and is defined as more than three months of pain related to a surgical region caused by a surgery when other causes of pain have been excluded. The causes or mechanisms of CPSP are not clear yet. However, it has been confirmed that CPSP is a result of postsurgical acute phase pain and that the intensity of the postsurgical acute phase pain is a reliable predictive indicator of CPSP onset. Therefore, it could be predicted that acute pain control after anesthesia and surgery could affect the onset of CPSP, but there are no sufficient studies regarding this.

Therefore, we planned this study, assuming that systemic lidocaine and magnesium administration during anesthesia and surgery could affect the incidence rate of not only postmastectomy acute pain but also the chronic pain, PMPS.

**4. Objectives**

The primary objective of this study was to investigate whether systemic administration of lidocaine during anesthesia of patients undergoing mastectomy affect postmastectomy acute pain. The degree and pattern of the pain were investigated with the pain NRS (NPI) and short form McGill pain questionnaire (SF-MPQ) 24 hours after a mastectomy. The secondary objective of this study was to investigate the existence, degree, and pattern of chronic pain three months after a mastectomy, which was assessed also with the SF-MPQ.

**5. Sample size calculation**

In a previous study in which postsurgical acute and chronic pains decreased in patients undergoing mastectomy by using dexmedetomidine during the surgery, the difference in the average SF-MPQ points between acute pain and chronic pain was 0.82 (Standard deviation was 1.12 and 0.84, respectively). When setting the non-inferiority margin of the SF-MPQ as 1 and α = 0.025 and β = 0.9, the calculated number of samples was 37 subjects in each group. When considering a non-completion rate of 15%, the calculated number of subjects was 86.

**6. Inclusion and exclusion criteria**

**(1) Inclusion criteria:** 20 to 65-year-old female patients who were planning to undergo a mastectomy whose American Society of Anesthesiologist (ASA) Physical Status Class was 1 or 2 and whose native language was Korean.

**(2) Exclusion criteria:** Patients who had an underlying pain from any causes or patients who were taking an analgesic; Patients who had diabetes or a severe heart, kidney, or liver disease, or a psychiatric disorder or a neurological disorder or were pregnant, Patients who had a contraindication or an allergy to the use of lidocaine

**7. The recruitment, enrollment and procedure of obtaining consent**

The study subjects were provided explanation on the purpose and method of the study at the pre-anesthesia examination room (clinic) or pre-operative visit one day before the surgery. Consent for study participation was obtained. Explanation and obtaining consent is performed in an independent area, and they are offered sufficient time to make the decision on study participation. Subject’s will to continue participating in the study will be orally confirmed at each of the 6 visits (one day before surgery, pre-anesthesia room, operation room, recovery room, 1 day after surgery, and 2 days after surgery) during the study period.

**At vulnerable subject recruiting, protective measures:** study subjects who are susceptible to compelling force or inappropriate influence, study subjects who have no decision-making capacity (pregnant women, pediatrics or adolescent, people with no decision-making capacity, and people whose decision-making process in voluntary participation is readily affected) are not involved in the clinical trial or are excluded from selection.

**8. Study design and methods**

This study was a prospective, randomized, double-blind, controlled study. The patients who planned to undergo a mastectomy were given an explanation about the purpose and method of the study on the day before the surgery in a counseling room of a ward or in a counseling room in an outpatient operation room. Then, written consent on participating in the study was given on the day of the surgery only from the patients who understood and agreed to the study. One researcher acquired the written consents and assessed the pain status before and after the surgery, and that researcher was not involved in the intraoperative anesthesia and the administration of the investigational drug and not aware of the group to which the individual subjects belonged to. On the morning of the day on which each patient was scheduled for mastectomy, using a random number sequence created by an internet website (http://www.random.org), the patients were randomly allocated to one of three groups in a 1:1:1 ratio: the lidocaine group (group L, n = 42), the magnesium group (group M, n = 42), or the control group (group C, n = 42). A bolus dose of the studied drug was administered for 15 minutes immediately after the subject was brought into the operating room, and the vital signs were checked from the beginning of anesthesia induction. Subsequently, a maintenance dose of the study drug was continuously administered through the intravenous route intraoperatively and was later stopped just before transferring the subject to a recovery room after surgery. Lidocaine (lidocaine hydrochloride) and magnesium (magnesium sulfate) were administered at 2 mg/kg and 20 mg/kg, respectively, for 15 minutes immediately after induction, followed by infusion at 2 mg/kg/h and 20 mg/kg/h infusion, respectively. The safety and analgesic effect of the lidocaine dose used in this study had already been demonstrated in previous studies. [17] The patients in the group C were administered and infused with the same volume of saline. Saline was added to the calculated dose to a total volume of 50 ml, and the treatments were labeled as “study drug” to ensure the double-blind administration. The concentration of serum magnesium was checked immediately before starting the study drug infusion and 1 hour after the infusion was stopped in all study groups.

The anesthesia and recovery of all the subjects were carried out by another researcher who was not aware of the allocated groups and not involved in the assessment before and after the surgery. Except for the administration of the investigational drug, the anesthesia was managed in the same manner as a general systemic anesthesia. When a subject arrived at the operation room, an electrocardiograph and a pulse oximetry were attached to the subject, and his/her blood pressure was measured. Propofol 1.5~2 mg/kg and remifentanil 1 µg/kg were intravenously injected for induction, and rocuronium 0.6 mg/kg was injected as a muscle relaxant followed by endotracheal intubation. The anesthesia was maintained with 50% oxygen, desflurane, and remifentanil. The bispectral index was monitored to maintain an appropriate anesthetic depth, and the concentration of the administered desflurane was controlled to maintain the bispectral index score in a range between 40 and 60. In addition, the administration rate of remifentanil was controlled so that the intraoperative blood pressure and heart rate could be maintained within 20% of the basal blood pressure and heart rate of the subject. Thirty minutes before the expected end point of the surgery, propacetamol 2 g was intravenously injected to control postsurgical pain along with palonosetron 0.075 mg to prevent nausea and vomiting. When the surgery was finished, the administration of desflurane and remifentanil was stopped, and glycopyrrolate 0.2 mg and neostigmine 1 mg were used to reverse the muscle relaxant. When the subject’s consciousness returned and respiration recovered, extubation was performed, and then, the subject was transported to the recovery room. The patients’ management in the recovery room and their postoperative management were done according to general instructions. However, it was necessary to monitor for abnormal reactions until the action time of the lidocaine and magnesium ended as part of the recovery room and postoperative management of the patient. Therefore, to monitor for abnormal reactions and toxic effects related to lidocaine and magnesium, from the time after the anesthesia was finished and the lidocaine administration was stopped, the patients’ vital signs such as heart rate, respiratory rate, and oxygen saturation were monitored, and the patients’ pronunciation was checked to determine whether they were inarticulate or their consciousness had changed. If there were no abnormal reactions after more than 30 minutes during patient monitoring, the patient was discharged from the recovery room.

**9. Variables**

(1) Other researcher who doesn’t know about patient’s randomization visits patients before and 1 day after surgery and performs QoR-40 survey. The quality of recovery of patients after general anesthesia is evaluated with global scores (total 200 points) including subtotal scores about 5 items such as Emotional status, Physical comfort, Physiological support, Physical independence, and Pain. The primary end point of this study is QoR 40 24 hours after surgery, and more than 10 points difference of the mean between two groups was considered as significant difference.

(2) Vital signs initially measured prior to anesthesia induction were recorded.

(3) The total amount of remifentanil and the mean concentration of desflurane are recorded.

(4) After the end of surgery,

① The total operation time was defined as the time from the beginning and end of the operation, and total anesthesia time was defined as the time from anesthesia induction to anesthesia end time.

② In order to measure the recovery rate, the time from the end of administration of main anesthetic agent to the response to verbal comments as well as BIS score, and the time taken till the extubation of endotracheal tube as well as BIS score are recorded. Vital signs are also evaluated for each time.

③ Bradycardia of less than 50/minute during anesthesia, hypotension of MAP < 60 mmHg, and used medications under anesthesia are recorded. Other problems are evaluated and other used medications are also recorded.

(6) After the entrance of recovery room,

① The degree of pain of patients is evaluated using Numeric Pain Intensity Scale (NPIS) on 0-10 scale at admission and discharge, and greater pain is higher score.

② The analgesics and anti-emetics used in the recovery room are recorded and the time to stay in the recovery room, from the entrance to discharge, is recorded.

③ Administered medications as well as other symptoms that patients complained have except pain or nausea/vomiting are recorded.

(7) Sedation score was measured at admission and discharge of recovery room using 5-point scale. [18] Sedation status of patients is evaluated on a scale of 1-5; 1 point- completely awake, 2 point- little drowsy, 3 point- seemingly sleeping but responding to a noise, 4 point- sleeping signs and slow response to a noise, and 5 points-sleeping with response to painful stimuli but without response to a voice.

(8) At the time to perform QoR 40 24 hours after surgery, a type of analgesics administered and total dose are recorded. Also, at the time of QoR 40 after surgery, the number as well as the degree of nausea/vomiting and the dose of anti-emetics are recorded, and the hospital stay of examinees from surgery to discharge also need to be found and recorded.

(9) The evaluation of the degree and pattern of acute pain for 24 hours after the end of surgery – Numeric Pain Intensity scale (NPI scale), short form McGill pain questionnaire (SF-MPQ)

(10) The absence or presence of post-operative pain 1 month after surgery, if present, the evaluation of the location and pattern of pain: SF - MPQ

(11) The absence or presence of post-operative pain 3 months after surgery, if present, the evaluation of the location and pattern of pain: SF - MPQ

(12) The management of missing data: This study greatly relies on patients’ compliance as including patient survey; the data of corresponding patients are discarded in case of missing data.

**10. Statistical analyses**

Results consisting of continuous data following a normal distribution were expressed as the mean ± standard deviation while results consisting of continuous data that did not follow a normal distribution were expressed as the median (interquartile range). Categorical data were expressed as the number of patients (ratio or %). Depending on the characteristics of the data, a continuous variable was analyzed by an independent t-test (or Mann-Whitney rank sum test); a nominal variable was analyzed by a chi-square (or Fisher’s exact test), and the temporal variation of a continuous variable was analyzed by repeated measures ANOVA with the Bonferroni correction. Data were considered as significant when the p-value was smaller than 0.05. The statistical analysis was performed with PASW statistics 20 (SPSS Inc., USA).

**11. Withdrawal**

(1) Patients who refuse to participate in the study; even when we have the consent form, we will check whether participants would like to participate in this research when we visit them before/after anesthesia, meaning they can tell us anytime if they have no intention to participate.

(2) Patients who must undergo a repeat operation because of complications related to the initial operation or a positive surgical margin.

**12. Predictable side effects and precautions**

**Lidocaine** can rarely cause allergic reactions such as itchiness and rash, as well as nausea and vomiting. Also, there can be toxic effect(s) in overdose [19], and the toxic effects of Lidocane are known to include dizziness or tongue numbness at the serum concentration of 5-10 µg/mL, lack of consciousness or seizure at 10-15 µg/mL, and coma at the concentration greater than 15 µg/mL. However, these toxic effects occur at the serum concentration greater than 5 µg/mL and lidocane 400 mg needs to be administered at once. However, in previous studies, which measured the serum concentration of lidocaine at different time points while administering similar dosage of lidocaine as in this study, no results were greater than 5 µg/mL. [20] The monitoring plan for lidocaine-related adverse effects and toxic effects includes checking speech impairment or change of consciousness while monitoring of vital signs of patients such as ECG, respiratory rate, and oxygen saturation once the lidocaine administration is stopped after the end of anesthesia, and checking abnormality on the post-operative day 1 and 2. The standard treatment plan about lidocaine adverse effects is closed monitoring, and anti-seizure medication is administered in case of seizure due to severe toxic effect. Also, rapid airway management and respiratory support should be performed in case of respiratory failure caused by loss of consciousness or coma.

**Magnesium** can cause gastrointestinal symptoms such as nausea, vomiting, and diarrhea, and toxic effects can occur when serum magnesium concentration is greater than 4 mEq/L. Common toxic effects include prolonged muscle relaxation, intensified sedative effect, and severe bradycardia or hypotension in patients with cardiovascular disease. [21-23] However, in previous studies which administered similar dosage to this study, the serum concentration was not greater than 4 mEq/L, and no severe adverse effect(s) were reported. The monitoring plan for magnesium-related adverse effects and toxic effects includes checking vital signs of patients in the recovery room once the magnesium administration is stopped as well as the patellar tendon reflex to examine muscle contraction. Also it includes checking the absence or presence of abnormality on the post-operative day 1 and 2. Also, serum concentration of magnesium is measured 2 hours after stopping the administration of experimental medication(s). Therapeutic regimens about hypermagnesemia and toxic effect include intravenous injection of calcium gluconate to antagonize neuromuscular function and cardiovascular effects, or reducing magnesium level by administrating fluid and furosemide diuretics. If respiratory failure occurs due to the weakness of respiratory muscle caused by severe toxicity, airway management and respiratory support may be required.

**13. The evaluation of safety**

For all study subjects, hemodynamic stability and appropriate ventilation is evaluated under the supervision of a trained physician in the department of anesthesia and pain medicine prior to anesthesia induction, maintaining anesthesia, and to the recovery room after extubation. Also, they receive treatment such as cardiovascular medications, respiratory support, and increase fraction of inspired oxygen according to the physician’s decision. In case of unpredictable medication adverse effect(s) including allergic reactions, appropriate treatment should be provided immediately and is to be reported to the IRB within 1 week. Also, in case that one or more of the following suspected unexpected serious adverse reactions (death, life threatening, hospitalization or prolongation of hospitalization, constant or meaningful disability or impaired function, medically significant), the investigator(s) and the sponsor must report to the IRB within 24 hours after awareness and report to the Ministry of Food and Drug Safety (MFDS) in the incidence of SUSAR. All information of the serious adverse event must be written with additional follow up information on the SUSAR report form. The copy of the report must be stored with the subject’s CRF.

**14. The plan for the interim analysis and range of early termination of the study**

Intermittent analysis is not planned, and the study can be completed when 126 patient data are collected.

**15. The continuous monitoring of drug safety and procedure**

The principle investigator (an authorized investigator in the absence of the principle investigator) is designated as personnel for data security, and warrants the completeness of the data by collecting and reviewing the data monthly and monitoring about constant safety, and establishes the safety of the study subjects.

**16. The storage of materials and confidentiality**

The study data will be used only for the purpose of this study. Access and analysis of the data will be restricted to the principle investigator and the researchers. Identifiable information of the clinical study subjects will be coded and recorded separately from the screening sheet. Data will be stored in a locked cabinet as well as access-limited computer.

**17. The schedule of research**

(1) The planning of research and certification of IRB: May 1. 2014 ~ June 30. 2014

(2) Data collection: 1 year within IRB certification

(3) Statistical performance and analysis of study results: 2 months within the collection of subjects

(4) Writing the article: 3 months after analyzing of results

**18. Ethics and regulation**

1) This study protocol conformed to the ethical guidelines of the 1975 Helsinki Declaration and International Conference on Harmonization of Technical Requirements of Pharmaceuticals for Human Use (ICH) Note for Guidance on Good Clinical Practice (ICH, Topic E6, 1995)

2) This study was approved by the Institutional Review Board of Severance Hospital.

3) Compensation: There is no financial compensation for patients who participate in this study.

**19. References**

1. Wu CL, Raja SN: **Treatment of acute postoperative pain**. *Lancet* 2011, **377**(9784):2215-2225.

2. Alves Nogueira Fabro E, Bergmann A, do Amaral ESB, Padula Ribeiro AC, de Souza Abrahao K, da Costa Leite Ferreira MG, de Almeida Dias R, Santos Thuler LC: **Post-mastectomy pain syndrome: incidence and risks**. *Breast* 2012, **21**(3):321-325.

3. Meijuan Y, Zhiyou P, Yuwen T, Ying F, Xinzhong C: **A retrospective study of postmastectomy pain syndrome: incidence, characteristics, risk factors, and influence on quality of life**. *ScientificWorldJournal* 2013, **2013**(1537-744X (Electronic)):159732.

4. Fecho K, Miller NR, Merritt SA, Klauber-Demore N, Hultman CS, Blau WS: **Acute and persistent postoperative pain after breast surgery**. *Pain Med* 2009, **10**(4):708-715.

5. Jain G, Bansal P, Ahmad B, Singh DK, Yadav G: **Effect of the perioperative infusion of dexmedetomidine on chronic pain after breast surgery**. *Indian J Palliat Care* 2012, **18**(1):45-51.

6. Fawcett WJ, Haxby EJ, Male DA: **Magnesium: physiology and pharmacology**. *Br J Anaesth* 1999, **83**(2):302-320.

7. Kehlet H, Wilmore DW: **Multimodal strategies to improve surgical outcome**. *Am J Surg* 2002, **183**(6):630-641.

8. Koppert W, Weigand M, Neumann F, Sittl R, Schuettler J, Schmelz M, Hering W: **Perioperative intravenous lidocaine has preventive effects on postoperative pain and morphine consumption after major abdominal surgery**. *Anesth Analg* 2004, **98**(4):1050-1055, table of contents.

9. Vigneault L, Turgeon AF, Cote D, Lauzier F, Zarychanski R, Moore L, McIntyre LA, Nicole PC, Fergusson DA: **Perioperative intravenous lidocaine infusion for postoperative pain control: a meta-analysis of randomized controlled trials**. *Can J Anaesth* 2011, **58**(1):22-37.

10. De Oliveira GS, Jr., Fitzgerald P, Streicher LF, Marcus RJ, McCarthy RJ: **Systemic lidocaine to improve postoperative quality of recovery after ambulatory laparoscopic surgery**. *Anesth Analg* 2012, **115**(2):262-267.

11. Albrecht E, Kirkham KR, Liu SS, Brull R: **Peri-operative intravenous administration of magnesium sulphate and postoperative pain: a meta-analysis**. *Anaesthesia* 2013, **68**(1):79-90.

12. Myles PS, Hunt JO, Nightingale CE, Fletcher H, Beh T, Tanil D, Nagy A, Rubinstein A, Ponsford JL: **Development and psychometric testing of a quality of recovery score after general anesthesia and surgery in adults**. *Anesthesia and analgesia* 1999, **88**(1):83-90.

13. Myles PS, Weitkamp B, Jones K, Melick J, Hensen S: **Validity and reliability of a postoperative quality of recovery score: the QoR-40**. *Br J Anaesth* 2000, **84**(1):11-15.

14. Murphy GS, Szokol JW, Greenberg SB, Avram MJ, Vender JS, Nisman M, Vaughn J: **Preoperative dexamethasone enhances quality of recovery after laparoscopic cholecystectomy: effect on in-hospital and postdischarge recovery outcomes**. *Anesthesiology* 2011, **114**(4):882-890.

15. De Oliveira GS, Jr., Ahmad S, Fitzgerald PC, Marcus RJ, Altman CS, Panjwani AS, McCarthy RJ: **Dose ranging study on the effect of preoperative dexamethasone on postoperative quality of recovery and opioid consumption after ambulatory gynaecological surgery**. *Br J Anaesth* 2011, **107**(3):362-371.

16. Buchanan FF, Myles PS, Cicuttini F: **Effect of patient sex on general anaesthesia and recovery**. *Br J Anaesth* 2011, **106**(6):832-839.

17. Kuo CP, Jao SW, Chen KM, Wong CS, Yeh CC, Sheen MJ, Wu CT: **Comparison of the effects of thoracic epidural analgesia and i.v. infusion with lidocaine on cytokine response, postoperative pain and bowel function in patients undergoing colonic surgery**. *British Journal of Anaesthesia* 2006, **97**(5):640-646.

18. Paris A, Kaufmann M, Tonner PH, Renz P, Lemke T, Ledowski T, Scholz J, Bein B: **Effects of clonidine and midazolam premedication on bispectral index and recovery after elective surgery**. *Eur J Anaesthesiol* 2009, **26**(7):603-610.

19. Liu S: **Local Anesthetics: Clinical Aspects**. In: *Essentilas of Pain Medicine and Regional Anesthesia.* Edited by HT B, SN R, RE M, SS L, SM F. Philadelphia, PA, USA: Elsevier, Churchill Livingstone; 2005: 558-565.

20. McCarthy GC, Megalla SA, Habib AS: **Impact of intravenous lidocaine infusion on postoperative analgesia and recovery from surgery: a systematic review of randomized controlled trials**. *Drugs* 2010, **70**(9):1149-1163.

21. Fuchs-Buder T, Wilder-Smith OH, Borgeat A, Tassonyi E: **Interaction of magnesium sulphate with vecuronium-induced neuromuscular block**. *Br J Anaesth* 1995, **74**(4):405-409.

22. Vissers RJ, Purssell R: **Iatrogenic magnesium overdose: two case reports**. *J Emerg Med* 1996, **14**(2):187-191.

23. Kiran S, Gupta R, Verma D: **Evaluation of a single-dose of intravenous magnesium sulphate for prevention of postoperative pain after inguinal surgery**. *Indian J Anaesth* 2011, **55**(1):31-35.
